# Symptomatic SARS-CoV-2 breakthrough infections broaden the repertoire of Spike-reactive CD4 T cells

**DOI:** 10.1128/mbio.03549-25

**Published:** 2025-12-12

**Authors:** Emil Johansson, Yeji Lee, Vicente Fajardo-Rosas, Adam Abawi, Ashmitaa Logandha Ramamoorthy Premlal, April Frazier, Jason A. Greenbaum, Pandurangan Vijayanand, Ricardo da Silva Antunes, Alessandro Sette

**Affiliations:** 1 Center for Infectious Disease and Vaccine Research, La Jolla Institute for Immunology (LJI), La Jolla, California, USA; 2 Department of Experimental Medical Science, Lund University 5193, Lund, Sweden; 3 Bioinformatics and Systems Biology Graduate Program, University of California, San Diego (UCSD) 8784 , La Jolla, California, USA; 4 Department of Medicine, Division of Infectious Diseases and Global Public Health, University of California, San Diego (UCSD) 8784 , La Jolla, California, USA; Tsinghua University, Beijing, China

**Keywords:** T cell, SARS-CoV-2, vaccines, T-cell repertoire

## Abstract

SARS-CoV-2 breakthrough infections (BTIs) are relatively common, but little is known in terms of differentiating responses associated with asymptomatic BTI (ABTI) versus symptomatic BTI (SBTI). Here, we investigated the impact of ABTI and SBTI on antibody and T-cell responses toward Spike. SBTI donors had significantly higher plasma anti-Spike RBD IgG titers compared to both ABTI and SARS-CoV-2 vaccinated donors with no signs of previous infection (VAX) donors. While no impact of ABTI or SBTIs was found in the magnitude of Spike-specific CD4 and CD8 T-cell responses, both ABTI and SBTI donors had significantly higher CD4 T-cell responses toward non-Spike antigens. In-depth characterization of Spike-specific CD4 and CD8 T cells at scRNA/TCRseq level revealed that ABTI and SBTI induced different alterations of the CD4 compartment. These included an *IFNG^high^*-skewed response among cytokine-producing cells and a concurrent expansion of type II IFN-responsive T_H_17-like cells in SBTI. SBTI donors were further found to have an increased CD4 TCR repertoire diversity compared to VAX donors. ABTI-associated alterations of the Spike-specific compartment were intermediate between SBTI and VAX groups, likely reflecting lower antigen exposure and less inflammatory environment during ABTIs versus SBTIs.

**IMPORTANCE:**

SARS-CoV-2 mRNA vaccines have been shown to induce robust T-cell responses, crucial for long-term protection against severe SARS-CoV-2 infection. Hybrid immunity, created by a combination of vaccination and infection, has been associated with improved protection against severe SARS-CoV-2 infection. Here, we have investigated the impact of asymptomatic and symptomatic breakthrough infections (BTIs) on T-cell responses toward Spike, compared to donors that have only received mRNA SARS-CoV-2 vaccines. Symptomatic, and to a lesser extent asymptomatic, BTIs broadened the repertoire of Spike-reactive CD4 T cells and induced a more pro-inflammatory Spike-reactive T-cell response capable of enhancing activation of TH17-like cells. These findings represent the first characterization of T-cell responses in ABTI in comparison to SBTI, and in comparison to vaccinated individuals (VAX) who did not experience BTIs.

## INTRODUCTION

SARS-CoV-2 breakthrough infections (BTIs) have become relatively common, driven by viral evolution that enables immune escape from B- and T-cell responses induced by previous vaccinations and infections, and waning of vaccine- or previous infection-induced immunity over time (1–4). BTIs may function as natural boosters that broaden and renew the immune protection provided by vaccination or previous infection and are recognized as playing an important role in maintaining an “immunity wall” against severe COVID-19 and its complications (4–7).

BTIs have been generally characterized in terms of magnitude and breadth of cross-neutralizing antibody responses (8–10), durability of T-cell immunity across SARS-CoV-2 variants (11–14), and the complex interplay between vaccination timing, viral load, and immune response (15–17). BTIs boost T-cell responses, broaden the range of antigens targeted by cellular responses beyond the Spike (S) antigen (12, 15, 18, 19), and generate novel T-cell epitopes that recognize mutations found in Delta and Omicron variants (19–21).

It is commonly observed that BTIs encompass a wide spectrum of clinical presentations, ranging from symptomatic disease (symptomatic BTIs; SBTIs) to completely asymptomatic (asymptomatic BTIs; ABTIs) (22, 23). However, the immunological profiles of T cells responding to antigen stimulation as a function of SBTI versus ABTI have not been described. The different inflammatory environments associated with BTIs may also drive distinct T-cell differentiation programs, underlying differences in T-cell subset activation, functional programming, and effector capabilities that remain poorly characterized at the single-cell level (24, 25). We have previously shown that ABTI was associated with an expansion of T_H_17-like cells compared to vaccinated individuals without infection (VAX) (26), but it is not known how these cells are impacted by SBTI. Further research is therefore needed to elucidate whether this represents a protective immune signature that prevents progression to SBTI, or whether the expansion reflects inflammatory events occurring during ABTI that would be amplified in SBTI.

Activation-induced marker (AIM) assays have become a standard method for measuring and characterizing antigen-specific T-cell responses, including those against SARS-CoV-2, due to their HLA-agnostic and function-independent attributes (27–29). It was recently reported that certain T-cell subsets, including T_H_17 and T_reg_ cells, can undergo TCR-independent activation by cytokines during antigen stimulation, leading to non-specific upregulation of activation markers (11, 30). However, “bystander” populations may themselves provide important biological insights into vaccine or infection *in vivo* responses and reactogenicity (31, 32).

Here, we used single-cell RNA sequencing (scRNAseq) and T-cell receptor sequencing (scTCRseq) to characterize Spike (S)-specific T-cell responses in VAX, ABTIs, and SBTIs, to define distinct immunological signatures related to T-cell functionality and T-cell subsets responding to antigen stimulation. We found that SBTIs broadened the repertoire of S-reactive CD4 T cells and induced an *IFNG^high^*-skewed pro-inflammatory T_H_1 response that enhanced the activation of S-specific T_H_17-like cells.

## RESULTS

### BTI increases antibody titers and T-cell response breadth

Our goal was to investigate the impact of SARS-CoV-2 SBTI on the antibody quantity and on the quantity and quality of T-cell responses, in comparison to responses observed in ABTI and VAX donors. Accordingly, we assembled a study cohort of COVID-19 vaccinated donors (Table 1) which included 13 donors who had reported a SBTI and compared them to 15 donors who had experienced an ABTI and 18 VAX donors, both selected from a previous study (26). The VAX and ABTI donors consistently tested negative in all PCR or antigen SARS-CoV-2 diagnostic tests that they undertook and remained asymptomatic for an average of 199 and 187 days, respectively (26). Donors were defined as having experienced ABTI on the basis of having T-cell responses toward non-S antigens, as detected using a peptide “megapool” (MP) of experimentally validated SARS-CoV-2 non-S epitopes (CD4RE) with minimal homology to endemic common cold coronaviruses (26, 33). Conversely, VAX donors had no detectable reactivity to the CD4RE pool using a stringent stimulation index (SI) cutoff (26, 33).

**TABLE 1:** Donor characterization^*a*^

	VAX (*n* = 18)	ABTI (*n* = 15)	SBTI (*n* = 13)
Sex (male/female)			
Male	8	4	2
Female	10	11	11
Age (mean ± SD)	52 ± 18	43 ± 15	42 ± 16
Days since last vaccine (mean ± SD)	129 ± 59	133 ± 81	158 ± 98
Days without symptoms (mean ± SD)	199 ± 184	187 ± 155	NA
Days since symptom onset (mean ± SD)	NA^*b*^	NA	103 ± 98
Vaccine doses (*n*)			
2	3	4	3
3	9	7	7
4	6	4	3
Sample year			
2021	3	4	0
2022	11	11	11
2023	4	0	2
Ethnicity			
White	15	11	12
Other	3	4	1

^*a*^ ABTI, asymptomatic breakthrough infection; SBTI, symptomatic breakthrough infection; VAX, vaccinated donors with no evidence of prior SARS-CoV-2 infection.

^*b*^ NA, not applicable.

Donors were matched for age, number of vaccine doses, days since last vaccination, year of sample donation, and ethnicity (Table 1). Samples were collected on average 129, 133, and 158 days post last vaccination for VAX, ABTI, and SBTI groups, respectively. For SBTI donors, samples were collected at an average of 103 days after symptom onset. All SBTI donors had fully recovered from acute COVID-19 symptoms and tested PCR-negative for SARS-CoV-2 at the time of blood collection.

**Fig 1:**
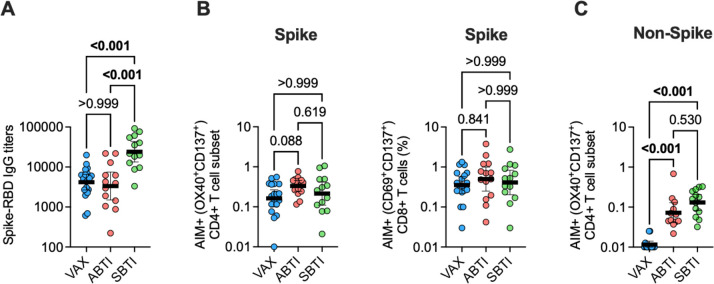
Symptomatic breakthrough infections (SBTIs) increase T-cell response breadth and plasma antibody titers. (**A**) Plasma levels of antibody titers of anti-Spike receptor-binding domain (RBD) in vaccinated donors with no evidence of prior SARS-CoV-2 infection (VAX, *n* = 18), asymptomatic breakthrough infection (ABTI, *n* = 15), and SBTI (*n* = 13) donors. (**B**) Spike-specific AIM^+^ CD4 (OX40^+^CD137^+^; left panel) and CD8 (CD69^+^CD137; right panel) T-cell responses in VAX (*n* = 18), ABTI (*n* = 14), and SBTI (*n* = 13) donors. (**C**) AIM^+^ CD4 T-cell responses toward non-Spike (CD4RE) SARS-CoV-2 antigens in VAX (*n* = 14), ABTI (*n* = 12), and SBTI (*n* = 13) donors. *P* values from two-tailed Kruskal-Wallis tests followed by Dunn’s multiple comparisons test, and geometric mean ± 95% confidence interval are shown.

As expected (19, 26), SBTI donors had significantly higher anti-S RBD IgG titers compared to both VAX (*P* < 0.001) and ABTI (*P* < 0.001) groups (Fig. 1A). In line with the previously described high stability of S-specific T-cell responses (26), the magnitude of S-reactive CD4 and CD8 T-cell responses was not significantly different between the three groups (Fig. 1B), as measured by the combined use of a MP of peptides spanning the entire sequence of the S antigen and an AIMassay (34). The magnitude of CD4 T-cell responses toward non-S antigens (CD4RE) showed the highest response in the SBTI group, followed by the ABTI group, with both groups being significantly higher compared to VAX donors (*P*<0.001) (Fig. 1C).

Taken together, these results show that while S-reactive T-cell responses are relatively stable in terms of magnitude and are not affected by BTIs, BTI increases breadth of SARS-CoV-2-reactive CD4 T cells, and SBTI increases the anti-S RBD IgG titers. Although not significant, responses to the non-S antigens were highest in SBTI donors compared to ABTI donors, possibly reflecting stronger or longer antigenic stimulation, or a more pronounced pro-inflammatory environment.

### Definition of S-reactive CD8 T-cell subsets associated with the three study groups

We hypothesized that differences in the inflammatory environment and antigenic stimulation associated with the different vaccination and infection exposures could also alter the phenotypes and quality of S-reactive T cells. We therefore sorted AIM^+^ CD8 T cells following S MP stimulation and performed scRNAseq and scTCRseq of the exclusive S-reactive cells (CD69^+^CD137^+^). Samples were obtained from the same individuals in the cohorts described above. In total, 89,839 CD8 T cells were included in the analysis following quality control (QC) assessment of the data set.

**Fig 2:**
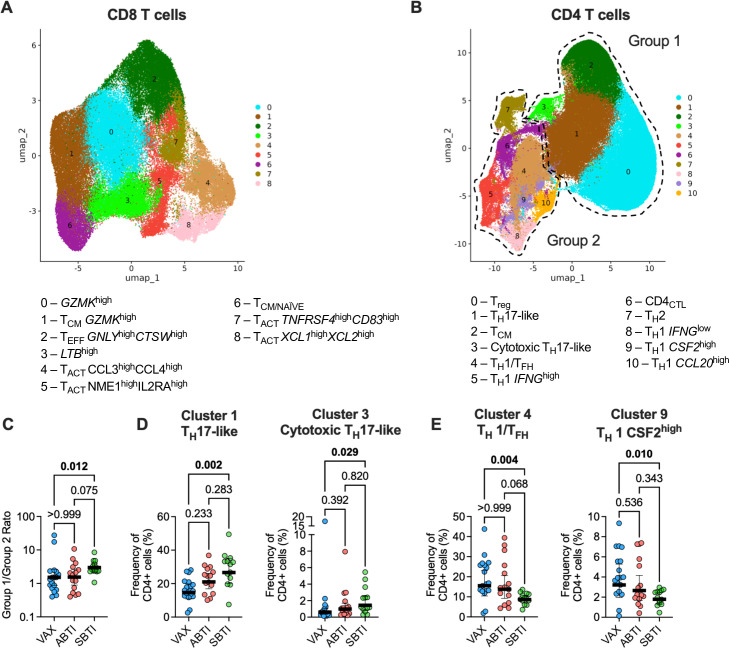
Single-cell characterization of Spike-specific T cells. UMAP projection of Spike-specific CD8 (**A**) and CD4 (**B**) T cells sorted from vaccinated donors with no evidence of prior SARS-CoV-2 infection (VAX, *n* = 18), asymptomatic breakthrough infection (ABTI, *n* = 15), and symptomatic BTI (SBTI, *n* = 13) donors. (**C**) Ratio of Groups 1 and 2 cells per donor. Frequency of T_H_17-like (left panel) and cytotoxic T_H_17-like (right panel) CD4 T-cell clusters (**D**), and T_H_1/T_FH_ (left panel) and T_H_1 CSF2^high^ clusters (right panel) (**E**). *P* values from two-tailed Kruskal-Wallis tests followed by Dunn’s multiple comparisons test, and median ± 95% confidence interval are shown.

Based on their gene expression and shared nearest neighbor (SNN)-based cluster analysis, we identified nine CD8 T-cell clusters (Fig. 2A). Fig. S1A shows cluster marker gene expression, respectively, which was further visualized using UMAP (Fig.S1B). High expression of *GZMK* was found in the two most abundant clusters, Clusters 0 (*GZMK*^high^), and Cluster 1 (T_CM_ *GZMK*^high^) which further had high expression of central memory markers *TCF7*, *IL7R*, and *KLF2*. Cluster 2 (T_EFF_ *GNLY*^high^ *CTSW*^high^) contained effector cells with high *GNLY* and *CTSW* expression, and Cluster 3 (*LTB*^high^) cells were characterized by high expression of *LTB*. Cells in Clusters 4, 5, 7, and 8 were found to have high expression of genes previously reported to be upregulated following recent activation. Cells in Cluster 4 (T_ACT_ CCL3^high^CCL4^high^) expressed high levels of *CCL3* and *CCL4* (35), cells in Cluster 5 (T_ACT_ NME1^high^IL2RA^high^) expressed high levels of NME1 and IL2RA (36), cells in Cluster 7 (T_ACT_*TNFRSF4*^high^*CD83*^high^) expressed high levels of CD83 and TNFRSF4 (OX-40) (37), and cells in Cluster 8 (T_ACT_*XCL1*^high^*XCL2*^high^) expressed high levels of XCL1 and XCL2 (35). Cells in Cluster 6 (T_CM/NAÏVE_) had high expression of naïve and central memory markers *TCF7*, *LEF1*, and *SELL* (38).

### Phenotypes of S-reactive CD8 T cells are not altered by BTIs

We did not observe significant differences in the cluster frequency of CD8 T-cell subpopulations across the three groups (Fig. S1C). Conflicting findings have been reported regarding the induction of CD8 T-cell exhaustion following repeated SARS-CoV-2 vaccination and infection (19, 39–41). Accordingly, we generated an exhaustion signature score based on 62 genes previously linked to CD8 T-cell exhaustion (39) (Table S1). In line with our previous findings (19, 26), we did not observe increased expression of the exhaustion-associated signature following ABTI or SBTI (Fig. S1D).

We next investigated the impact of BTI on the CD8 TCR repertoire by comparing the diversity of CDR3 sequences from paired TCRα and TCRβ chains, based on Chao1, Gini-Simpson, D50 diversity metrics. Consistent with the similar CD8 cluster frequencies across the three groups, we did not observe any significant differences in TCR repertoire diversity (Fig. S1D).

Taken together, these results show that the subsets of S-reactive CD8 T cells, although phenotypically heterogeneous, are remarkably similar across study groups and are not significantly altered by BTIs. Likewise, S-reactive CD8 T cells are associated with similar TCR repertoire diversity.

### S-reactive CD4 T cells are phenotypically heterogenous

We performed scRNAseq and scTCRseq on S-reactive CD4 T cells by sorting AIM^+^ (OX40^+^CD137^+^) cells following S MP stimulation. After applying similar QC measures as for CD8 T cells, 140,640 CD4 T cells were included in the analysis. In total, we identified 11 CD4 T-cell clusters (Fig. 2B), which were annotated on the basis of their differentially expressed genes (Fig. S2A) and further visualized using UMAP (Fig. S2B). Cluster 0 (T_reg_) cells showed upregulated expression of the canonical Treg markers *FOXP3* and *IKZF2* (Fig. S2A). Cluster 1 (T_H_17-like) cells were enriched for T_H_17 signature genes, including *DPP4*, *IL4I1*, and *CTSH* (Fig. S2A). Cluster 2 (T_CM_) contained cells with enrichment for central memory signature genes *TCF7*, *IL7R*, and *KLF2* (Fig. S2A). Cluster 3 (cytotoxic T_H_17-like) cells had high expression of T_H_17-associated genes, as well as the cytotoxic genes *GNLY*, *CCL5*, and *PRF1* (Fig. S2A). A T_H_1 signature was found in Clusters 3, 4, 5, and 8–10 (Fig. S2A), including *TBX21* (T-bet), *IL2*, and *TNF*. Cells in Cluster 3 (T_H_1/T_FH_) cells had low cytokine expression but high expression of ID3, a marker of memory CD4 T cells with the capacity to give rise to both T_H_1 and T_FH_ cells (42). Cells in Cluster 5 (T_H_1 *IFNG*^high^) expressed high levels of *IFNG*, *IL2*, and *TNF*. Cluster 6 (CD4_CTL_) contained cells with high expression of cytotoxic genes *GZMB*, *GZMA*, *NKG7*, and *CCL5* (Fig. S2A). Cluster 7 (T_H_2) contained cells with high expression of *IL4*, *IL5*, *IL13*, and the Th2 lineage master regulator *GATA3* (Fig. S2A). Within clusters 9 and 10, the expression of *CSF2* was highest in Cluster 9 (T_H_1 *CSF2*^high^) cells and the expression of *CCL20* in Cluster 10 (T_H_1 *CCL20*^high^) cells (Fig. S2A).

These results demonstrate that both CD4 and CD8 T-cell subsets responding to *in vitro* S MP stimulation display remarkable phenotypic heterogeneity. The various subsets overlap with those reported in prior studies conducted by our laboratory and other groups (26, 41, 43).

### SBTI induces phenotypic changes of S-reactive CD4 T cells

Based on cluster relationship at different clustering resolutions, the 11 CD4 T-cell clusters could be combined into two primary groups each accounting for approximately 33% and 66% of the total number of cells (Fig. S2C), respectively. One containing the T_H_17-like, cytotoxic T_H_17-like, T_CM_ and T_reg_ clusters (Group 1) and the second containing the remaining clusters of T_H_1, T_H_2, and T_CTL_ cells (Group 2). The Group 1 cluster (T_H_17-like, cytotoxic T_H_17-like, T_CM_, and T_regs_) was significantly enriched in frequency in SBTI compared to the VAX donors (*P* = 0.012; Fig. 2C). Within Group 1, the frequency of Clusters 1 (T_H_17-like) and 3 (Cytotoxic T_H_17-like) was significantly higher in SBTI donors (*P* = 0.002 and *P* = 0.029, respectively) compared to VAX donors (Fig. 2D; Fig. S2D). In Group 2, SBTI donors had significantly lower frequencies of Cluster 4 (T_H_1/T_FH_) and Cluster 9 (CSF2^high^) (*P*=0.004 and *P*=0.010, respectively) compared to VAX donors (Fig. 2E). ABTI donors showed frequencies of these four clusters that were intermediate between VAX and SBTI donors although no statistical significance was observed.

Taken together, these results show that while BTIs do not alter the magnitude of S-specific CD4 T-cell responses, BTIs alter the phenotype of the S-reactive T cells. These changes were strongest among the SBTI donors, with a trend for similar alterations among the ABTI donors.

### T_H_1-biased CD4 T-cell responses promote T_H_17-like cell expansion

Recent studies highlighted the interplay between cells *in vitro* following peptide stimulation (11, 32). These are mediated by cytokines and receptor-ligand interactions between conventional T cell (T_CONV_) subsets such as T_regs_ and T_H_1/T_FH_^11^, as well as between T_CONV_ and MAIT cells (32). As several cytokine genes were upregulated in Group 2 clusters (Fig. S2A), we hypothesized that the activation of Group 1 clusters might be enhanced by the activation of Group 2 cells.

DGE analysis of the two groups revealed distinct expression patterns. Group 2 was characterized by strong cytokine expression, with the top 10 differentially expressed genes including *IL2*, *IL3*, *IL13*, *CSF2*, *IL21*, *IFNG*, and *CCL20*. These genes are involved in the Reactome Gene Sets “Signaling by Interleukins.” In contrast, Group 1 showed elevated expression of genes involved in the GO Biological Process “Cellular response to cytokine stimulus,” including cytokine receptors (*IFNGR2*, *IL2RB*, and *IL2RG*) and Interferon-induced genes (*IFI6*, *IFIT1*, and *ISG15*) (Table S2; Fig. S3A). Of note, these two groups resemble two previously described modules of activated CD4 T cells identified following TCR engagement (36), one with high expression of TCR signaling genes and one with high type II interferon signaling genes (30, 36) (Fig. S3B).

**Fig 3:**
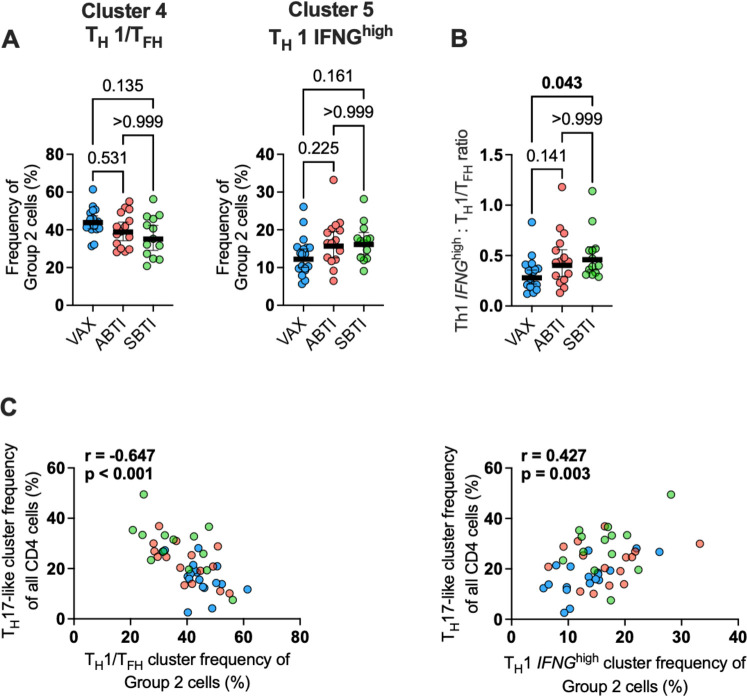
T_H_1 *IFNG*^high^ cells are positively associated with T_H_17 cell expansion. (**A**) Frequency of Cluster 4 (T_H_1/T_FH_) (left panel) and Cluster 5 (*IFNG*^high^) cells (right panel) among all Group 2 clusters. (**B**) Cluster 5 (*IFNG*^high^)/Cluster 4 (T_H_1/T_FH_) ratio for all donors. (**C**) Correlation between the frequency of T_H_17-like cells among all CD4 AIM^+^ cells with the frequency of T_H_1/T_FH_ (left panel) or T_H_1 *IFNG^high^* (right panel) cells among all Group 2 clusters. *P* values from two-tailed Kruskal-Wallis tests followed by Dunn’s multiple comparisons test or Pearson correlation test. Geometric mean ± 95% confidence interval are shown. Samples were collected from vaccinated donors with no evidence of prior SARS-CoV-2 infection (VAX, *n* = 18), asymptomatic breakthrough infection (ABTI, *n* = 15), and symptomatic BTI (SBTI, *n* = 13) donors.

We next examined the frequency of the clusters within Group 2 separately to determine how the frequency of these cells was associated with the expansion of Group 1 clusters. Interestingly, we observed an inverse association between Cluster 4 (T_H_1/T_FH_) and Cluster 5 (T_H_1 *IFNG*^high^). Specifically, we found a trend for decreased frequency of Cluster 4 (T_H_1/T_FH_) (*P* = 0.135) and increased frequency of Cluster 5 (T_H_1 *IFNG*^high^) (*P* = 0.161) (Fig. 3A), and a significantly higher T_H_1 *IFNG*^high^:T_H_1/T_FH_ ratio (*P* = 0.043) in SBTI donors compared to VAX donors (Fig. 3B). We observed a similar trend for higher T_H_1 *IFNG*^high^:T_H_1/T_FH_ ratio (*P* = 0.141) in the ABTI donors, in line with the moderate perturbation of Group 1 and Group 2 clusters observed in Fig. 2. We further found that the frequency of the T_H_17-like cluster was negatively associated with the frequency of the T_H_1/T_FH_ cluster (*r* = −0.647, *P* < 0.001) and positively associated with the frequency of the T_H_1 *IFNG*^high^ cluster (*r* = 0.427, *P* = 0.003) among all Group 2 clusters (Fig. 3C). No significant associations were found for T_CM_ or T_reg_ cells.

These results suggest that an *IFNG^high^*-T-cell response could promote the expansion of the T_H_17-like cells. Supporting this observation, cells from the T_H_17-like cluster in SBTI donors exhibited increased expression of genes involved in the Reactome Gene Sets “Interferon Signaling,” including *BST2*, *IFI6*, and *ISG15* (Fig. S3C; Table S3), in comparison to VAX donors. Taken together, these results highlight the interplay between subsets of S-reactive CD4 T cells, suggesting that TCR-dependent IFNγ release drives enhanced activation of type II IFN-responsive CD4 T cells (36).

### TCR profiles associated with the different donor cohorts

**Fig 4:**
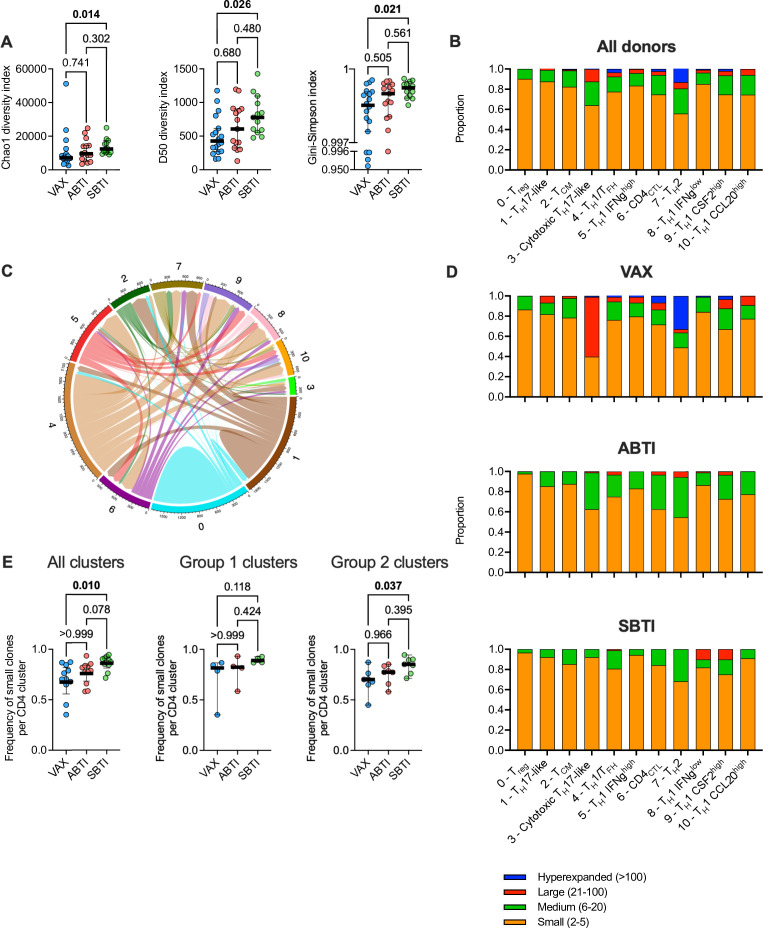
Symptomatic breakthrough infections (BTIs) broaden the repertoire of Spike-specific CD4 T cells. (**A**) Chao1, D50 (number of clones covering 50% or more of the repertoire), and Gini-Simpson diversity indexes. *P* values from two-tailed Kruskal-Wallis tests followed by Dunn’s multiple comparisons test, and median ± 95% confidence interval are shown. (**B**) Proportion of hyperexpanded, large, medium, and small clones in individual clusters. The number of cells was downsampled to an equal number of cells per cluster (**C**) Cord diagram displaying clonal sharing of all expanded clones (found in >1 cell) between clusters. Clones were identified based on paired alpha and beta CDR3 amino acid sequences. (**D**) Proportion of hyperexpanded, large, medium, and small clones in individual clusters for each of the three study groups. The number of cells was downsampled to an equal number of cells per cluster and group. (**E**) Frequency of small clones in all CD4 T-cell cluster (*n* = 11; right panel), in Group 1 clusters only (middle panel; clusters 1–3), or Group 2 clusters only (right panel; clusters 4–10). The number of cells was downsampled to an equal number of cells per study group. Samples were collected from vaccinated donors with no evidence of prior SARS-CoV-2 infection (VAX, *n* = 18), asymptomatic BTI (ABTI, *n* = 15), and symptomatic BTI (SBTI, *n* = 13) donors.

When comparing the TCR repertoire of all CD4 T cells, we found that SBTI donors had significantly higher Chao1 (*P* = 0.014) diversity index (Fig. 4A), reflecting an increased richness of the repertoire, that is, number of clones, driven by an increased proportion of singleton clones. In line with this, the D50 diversity index, defined as the number of clonotypes accounting for 50% or more of the total TCR repertoire, was significantly higher in SBTI compared to VAX donors (*P* = 0.026). Finally, the Gini-Simpson diversity index was significantly higher in SBTI donors compared to VAX donors (*P* = 0.021), highlighting a more even distribution of clonal sizes across the TCR repertoire. Taken together, these results indicate a reduced dominance of robustly expanded clones and increased frequency of weakly expanded and singleton clones following SBTIs. Similar to the phenotypic changes of S-reactive T cells, the characteristics of the TCR repertoire of the ABTI donors were intermediate to the VAX and the SBTI donors.

We next assessed clonal expansion and clonal sharing at the individual cluster level. We found expanded clones in all clusters, but in varying proportions. The lowest proportion of expanded cells was found in T_reg_ cells, while the largest proportion was found in T_H_2 cells (Fig. 4B). Extensive overlap between expanded clones was found across clusters, except for the T_reg_ cluster (Fig. 4C). Consistent with a recent publication (11), the T_reg_ cluster had the lowest proportion of clones shared with other clusters, and these cells potentially represent those activated in a bystander fashion by cytokines released from cells reactive to the S peptide pool. Examination of TCR clone expansion in individual clusters, downsampled to equal number of cells per cluster and donor group, revealed that expanded clones in ABTI and SBTI donors were predominantly small (2–5 cells) or medium (5–20 cells) sized, while the VAX donors also displayed large (20–100 cells) and hyperexpanded clones (>100 cells) (Fig. 4D). When comparing the proportion of small expanded clones in each cluster, we found that the SBTI donors had a higher proportion of small T-cell clones in all clusters compared to the VAX donors (*P* = 0.10; Fig. 4E, left). Importantly, the proportion of small expanded clones among Group 2 clusters remained significantly higher in SBTI compared to VAX donors (*P* = 0.037; Fig. 4E, right), but not for Group 1 clusters (*P* = 0.118; Fig. 4E, center). Taken together, these results show that SBTI infection increases the richness of the TCR repertoire of S-reactive CD4 T cells, associated with an increased frequency of small expanded clones. This increase was most prominent among the cytokine-producing Group 2 clusters, with non-significant trends observed among Group 1 clusters. This supports the conclusion that symptomatic BTI expands the bona fide Spike-specific TCR repertoire beyond what is achieved by vaccination alone. The characteristics of the TCR repertoire of the ABTI donors were intermediate to the VAX and the SBTI donors.

### Assessment of TCR specificity

**Fig 5:**
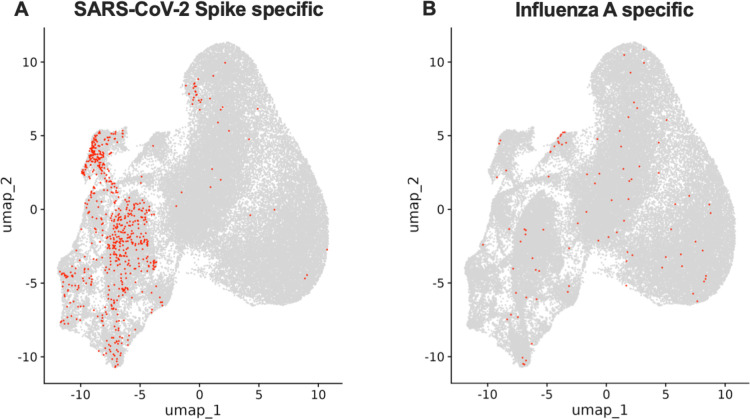
Lack of evidence for accumulation of non-Spike specific cells among Group 1 clusters. UMAP projection of cells with TCR alpha CDR3, V-segment, and J-segments matched with SARS-CoV-2 (**A**) and Influenza A (**B**) specific TCR sequences available at the VDJ database. Cells with matched TCR sequences are highlighted in red. Samples were collected from vaccinated donors with no evidence of prior SARS-CoV-2 infection (VAX, *n* = 18), asymptomatic breakthrough infection (ABTI, *n* = 15), and symptomatic BTI (SBTI, *n* = 13) donors.

A recent study suggested that T_H_17-like cells found among AIM^+^ cells were activated in a TCR-independent bystander fashion (30). To address this, we leveraged the VDJ database to identify S-specific TCRα chains (44). In total, we identified 668 S-specific cells, 647 of which were specific for the previously described immunodominant peptide TFEYVSQPFLMDLE (45, 46), present in our S MP (Table S4), representing 4.2% of all clonally expanded cells. In line with the recent publication (30), we found that these cells were primarily found among Group 2 clusters (Fisher’s exact test *P* < 0.001), with few cells observed across Group 1 T_H_17-like, cytotoxic T_H_17-like, T_CM_, and T_reg_ cells (Fig. 5A). To assess if the TCR clones in the Group 1 clusters represented cells activated through potential bystander activation, we next assessed if TCRα chains matched to previously described non-SARS-CoV-2 antigens in the VDJ database were accumulated in Group 1 clusters. However, we only found 168 cells with TCRα chain matches in the VDJ database (Fig. S3D; Table S5). The most common match was to Influenza A, a ubiquitous virus in the United States (47). Among all CD4 T cells, we found only 90 cells (0.1%) expressing previously defined Influenza A-specific TCRα, with no selective accumulation in Group 1 compared to Group 2 clusters (Fisher’s exact test *P* = 0.741; Fig. 5B), although an accumulation of Influenza A-specific TCRα was identified among cytotoxic T_H_17-like cells (Fisher’s exact test *P* < 0.001).

In conclusion, these results suggest that SBTIs broaden the repertoire of S-reactive T cells and promote an *IFNG*^high^ T_H_1 response that more efficiently activates S-reactive T_H_17-like cells.

## DISCUSSION

Here, we present the first characterization of T-cell responses in ABTI in comparison to SBTI and VAX donors. The characterization included determining the magnitude of responses and characterizing the functional T-cell subsets responding to antigen stimulation and associated TCR repertoires. The results indicate that ABTI responses are associated with characteristics that are intermediate between SBTI and VAX groups. The fact that SBTI responses are in general higher than ABTI indicates that the symptomatic nature of the BTI is not associated with an intrinsic defect of these donors to mount a T-cell response. In fact, individuals emerging from a SBTI episode, because of the higher level of immunity, might be associated (albeit temporarily) with higher levels of protection from further reinfection and symptomatic disease. Conversely, ABTIs are associated with intermediate levels of immune responses, which are lower compared to SBTIs. This might reflect lower levels of infection either in terms of viral titers or duration.

In line with previous studies (19, 26, 48), we found that the magnitude of S-reactive CD4 and CD8 T-cell responses was not significantly altered following BTIs, highlighting the stability of vaccine-induced T-cell responses. However, by employing scTCRseq, we found that following SBTIs, the S-reactive CD4 T cell repertoire is broadened by the emergence of smaller clones and reduced dominance of larger clones. In our previous study, we showed that BTIs induced *de novo* responses toward variant-specific epitopes (19), which could in part explain the observed broadening of the TCR repertoire in SBTI donors. In the case of ABTI donors, the median values of all three diversity measurements of ABTI donors were intermediate to that of VAX and SBTI donors, possibly due to reduced antigen exposure in these donors.

Further characterization of the S-reactive CD4 T cells revealed two main subsets, one containing cytokine-producing cells and a second group containing cytokine-responsive cells. SBTI donors were associated with an increased T_H_1 *IFNG*^high^:T_H_1/T_FH_ ratio among the cytokine producing cells, paralleling previous observations that hybrid immunity (SARS-CoV-2 vaccination and infection) increases frequencies of IFNγ-releasing CD4 T cells (41). Further, the frequency of *IFNG^high^* cells was positively associated with the expansion of type II IFN-responsive T_H_17-like cells. These results are in line with two major modules of memory CD4 T cells following TCR-mediated activation: one containing cytokine-producing cells, and a second containing cells whose activation was modulated through type II IFN signaling (36).

Consistent with the impact of IFNγ on T_H_17-like cells observed here, two recent studies showed that T-cell responses were augmented through a feedback loop where IFNγ, released by S-specific memory T cells, activated innate-like T cells (11), which in turn amplified the signal by releasing more IFNγ (32). This effect was dependent on the time between vaccination doses, with longer intervals being associated with reduced inflammatory signatures and higher recall-expansion of TCR clones observed in the previous vaccine dose (11). The increased T_H_1 *IFNG*^high^:T_H_1/T_FH_ ratio in SBTI donors observed here could therefore in part be reflective of a more recent exposure to SARS-CoV-2 compared to ABTI and VAX donors, combined with the heightened inflammatory state during a BTI (49). The bidirectional cross-talk between innate-like T cells and conventional T cells plays an important role during the mounting of an immune response toward vaccines and viruses (50, 51). The clear phenotypic alteration of the CD4 T cell compartment observed in SBTI suggests that the type II IFN-responsive cells also play an important role during a BTI, potentially in the orchestration of immune responses and protection from recurrent infection. However, it is still not known if these cells play a pro-inflammatory or an autoregulatory role. Here, T_regs_ were found to be the biggest type II IFN responsive cluster, but we did not observe any significant differences between the three study groups. The second largest cluster contained T_H_17-like cells with a gene signature, resembling pro-inflammatory T_H_ subsets linked to autoimmunity (52). However, we did not observe high levels of IL-17 family of cytokine genes or *IL22* in this cluster, and previous studies have not reported IL-17 to be an abundantly produced cytokine by S-reactive CD4 T cells (28, 53). More research is therefore needed to elucidate the role of the type II interferon responsive T_H_17-like cells observed here, and to determine if these cells represent bona fide T_H_17 memory cells, reflect a BTI-imprinted T_H_17-like transcriptional signature in S-reactive cells, or infection-primed S-specific cells developed during the BTI (49, 54). The T_H_17-like populations identified in our study (clusters 1 and 3) likely represent cells with T_H_17 characteristics representing transient activation states. The selective enrichment of these populations in SBTI compared to both VAX and ABTI likely reflects differences in the inflammatory environment and antigen exposure between natural infection and vaccination. During natural SARS-CoV-2 infection, prolonged systemic antigen exposure combined with tissue damage-associated inflammatory mediators (IL-6, IL-1β, and IL-23) may drive T_H_17 differentiation more effectively than the localized inflammation induced by mRNA vaccination (55). Whether the T_H_17-like populations represent beneficial barrier immunity to reinfections or contribute to symptomatic disease and inflammation requires further functional validation.

Surprisingly, we observed T_H_2 cell expansion in all three study groups, even though T_H_2 cells are not typically reported as a major subset of Spike-reactive T cells (26, 56). While T_H_2 responses are generally considered less protective against viral infections, T_H_2 expansion might represent a regulatory response to limit excessive T_H_1/T_H_17-mediated inflammation, and a recent study found enrichment of the expression T_H_2 cytokines in long-persisting CD4 T-cell clones (57), suggesting that these cells might play a role in the long-term protection against SARS-CoV-2.

Recent reports have raised concern that subpopulations of AIM^+^ cells, including T_H_17-like and T_reg_ cells, become activated through TCR-independent activation by cytokines (11, 30). Similar to a previous study (11), we observed that the majority of expanded TCR clones found in T_reg_ cells were found exclusively within this cluster, suggesting that these cells may indeed represent bystander-activated cells. However, the remaining clusters showed a high degree of clonal sharing between clusters. Corroborating recent findings (30), we observed that CD4 T cells specific for the S peptide TFEYVSQPFLMDLE were enriched among non-T_H_17-like cells. We therefore next investigated if we could find an accumulation of TCR clones previously described to be specific for non-SARS-CoV-2 antigens in the VDJ database among T_H_17-like cells, but no such enrichment of Influenza A-specific T cells was observed. However, it is important to note that this analysis is limited by the availability of TCR sequences from CD4 T cells at the VDJ database, which does not include TCR sequences specific for the ubiquitous virus EBV (58). We did not find sufficient evidence that the CD4 T cells with a cytokine-activated gene signature are Spike-specific CD4 T cells. However, as shorter antigen-exposure intervals have been shown to induce reduced expansion of recalled clones among AIM^+^ cells (11), the TH17-like cells could thus represent *de novo* responses to variant-specific epitopes following BTIs. The specificity of Group 1 cells could in future studies be addressed by sorting Group 1 cells and restimulating them with Spike peptides, or engineered T cell lines with TCRs from Group 1 cells could be generated and tested for Spike reactivity. Given the modest sample sizes of our study groups, the findings presented in this study should be considered exploratory and require validation in a larger cohort with improved statistical power. This is particularly important for confirming trends observed in comparisons between ABTI and SBTI, where differences were subtle and did not always reach statistical significance.

In conclusion, our data show that ABTI and SBTI are associated with different features of T-cell responses. This likely reflects the increased antigen exposure, reduced exposure interval, and heightened inflammatory environment during an SBTI compared to an ABTI, and in turn might influence the degree and duration of protection from further BTIs.

## MATERIALS AND METHODS

### Subjects and samples

All samples were collected by the Clinical Core at the La Jolla Institute and provided informed consent (26). Sample collection was approved by the LJI Institutional Review Board under IRB Protocol #VD-214. The VAX and ABTI donors were part of a longitudinal study where they were rigorously screened to ensure they tested negative for SARS-CoV-2 both before and during the sample collection period either by antigen or PCR test (26). The VAX and ABTI donors were selected to match the SBTI donors as best as possible in regards to gender balance, ethnicity, age, time since last vaccine dose, and number of vaccine doses (Table 1). Importantly, we found no significant differences between the three study groups in these parameters.

### Peripheral blood mononuclear cells (PBMC) and plasma isolation

Blood samples were collected in heparin-coated blood bags, and PBMCs were isolated by density-gradient sedimentation with Ficoll-Paque PLUS (Cytiva), as previously described (26, 59).

### SARS-CoV-2 S-RBD ELISA

Plasma titers of anti-S RBD IgG titers were determined by ELISA as described in previous studies (26, 60, 61).

### AIM assay

To study T-cell responses against SARS-CoV-2, two peptide MPs were prepared following the MP approach, previously outlined as a comprehensive method for analyzing T-cell responses across diverse epitopes and populations (34). A MP of 15-mer peptides overlapping by 10 spanning the entire S protein sequence (253 peptides) and a MP (CD4RE) composed of 284 experimental defined epitopes from non-S (R) region of SARS-CoV-2 (Table S4) were selected as previously described (28, 33).

To detect T-cell-specific responses, we employed an AIM assay methodology in combination with peptide pool stimulation using the dual activation marker expression of OX40^+^CD137^+^ or CD69^+^CD137^+^ to detect antigen-specific CD4 and CD8 T cells, respectively (34). Briefly, 1–2 × 10^6^ PBMCs were plated and immediately stimulated with MPs (1 μg/mL), or phytohemagglutinin-L (PHA) (10 μg/mL; Roche, San Diego, CA, USA) and DMSO as positive and negative controls, respectively, in RPMI 1640 supplemented in 5% human serum (Gemini Bio-Products) for 18–24 h. After culture, cells were collected, washed, and stained with Live/Dead eFlour 506 (Thermo Fisher), CD3 BUV805 (UCHT1, BD Biosciences), CD8 BV650 (RPA-T8, BioLegend, San Diego, CA, USA), CD19 V500 (HIB19, BD Biosciences), CD14 V500 (M5E2, BD Biosciences), CD4 BV605 (RPA-T4, BD Biosciences), OX40 PE-Cy7 (Ber-ACT35, BioLegend), CD137 APC (4B4-1, BioLegend), and CD69 PE (FN50, BD Biosciences) for 30 min at 4°C. All samples were acquired on a Bio-Rad ZE5 Analyzer (Bio-Rad Laboratories, Hercules, CA, USA) and analyzed with FlowJo 10.9 software (Tree Star, Ashland, OR, USA), as previously described (26).

The data were normalized with a minimum response level set at 0.005%. The specific T-cell responses were calculated by subtracting the background (DMSO stimulation) values. For each population, the limit of detection (LOD) was determined as the upper 95% confidence interval (CI) of the DMSO values, while the limit of sensitivity (LOS) was calculated as the median plus two times the standard deviation (SD) of DMSO. The SI was calculated as the percentage of stimuli response divided by the percentage of response in the DMSO control. Positive responses were defined as responses greater than LOS, with SI > 2 for CD4 T cells or SI > 3 for CD8 T cells. Responses with SI < 2 for CD4 T cells or SI < 3 for CD8 T cells were normalized to LOD. ABTI donors were identified by the first time point at which their CD4RE MP reactivity responses surpassed a highly stringent 10-fold SI threshold (26). While this cutoff provides good confidence in identifying individuals with previous SARS-CoV-2 exposure, the VAX donor group may still include individuals with previous exposure that did not generate detectable CD4RE responses.

### AIM^+^ T cell sorting and sample preparation

According to the previously described AIM assay protocol, PBMCs were thawed and stimulated for 24 h with the S MP. Cells were washed and stained with an antibody cocktail containing the antibodies described above for 30 min at 4°C, protected from light. TotalSeq-C oligonucleotide-conjugated antibodies (BioLegend) were also added at this step at 0.01 mg/mL final concentration (one distinct antibody per sample). After two washes in PBS, cells were resuspended in 500 μL of FACS buffer (PBS containing 2 mM EDTA (pH 8.0) and 0.5% BSA) and stored at 4°C until flow cytometry acquisition. S-specific CD4^+^ and CD8^+^ AIM^+^ T cells were subsequently sorted using a BD FACSAria Fusion cell sorter (Becton Dickinson), as previously described (26). On average, 20,000–40,000 AIM^+^ T cells per subject were sorted for single-cell RNA-seq assays (10x Genomics, Pleasanton). Cells were collected directly into low-retention, sterile 1.5 mL collection tubes (Thermo-Fisher) pre-chilled on ice and containing 500 μL of PBS:FBS solution (1:1, vol:vol) supplemented with RNAse inhibitor (1:100, Takara Bio).

### Cell isolation and single-cell RNA-seq and TCR-seq library preparation

A total of five to six different samples were multiplexed, each labeled with distinct TotalSeq-C DNA-oligo barcoded, totaling approximately 60,000 cells per 10× lane (equivalent to one well of the 10× chip). Samples were centrifuged at 600 × *g* for 10 min at 4°C, and the supernatant was carefully removed, leaving behind 10–12 μL of residual volume. Cell pellets were resuspended in 25 μL of 10x Genomics-compatible resuspension buffer (0.22 μm filtered PBS containing 0.04% ultrapure bovine serum albumin, Sigma-Aldrich). 33 μL of the resuspended cells was transferred to an 8-strip PCR tube for downstream processing as per the 10x Genomics protocol. The remaining cells were used for cell-counting QC.

Following the manufacturer’s recommendations, single-cell RNA libraries were generated using the 10x Genomics standard 5′TAG v2 chemistry. Both cDNA amplification and library preparation were carried out using 13 PCR cycles. Barcoded cDNA products were collected, quantified, and pooled at equimolar concentrations. The libraries were sequenced on the Illumina Novaseq6000 platform with paired-end sequencing (S4 100 × 100 cycles, Illumina) configured as follows: read length: read 1, 100 cycles; read 2, 100 cycles; i7 index, 10 cycles; and i5 index 10 cycles.

Size and quantity QCs were performed throughout the procedure by capillary DNA high sensitivity electrophoresis (HS NGS Fragment Kit, 1–6000 bp, Fragment Analyzer, Agilent) and Picogreen assay (Quant-iT PicoGreen dsDNA Assay Kits and dsDNA Reagents). Sequencing depth was aimed to reach 30,000 reads per cell for gene expression 8,000 reads/cell for TCR, and antibody-feature sequencing (multiplexing analysis).

### Single-cell transcriptome analysis

The Cellranger multi pipeline (v8.0.1) was used to perform alignment to the pre-built GRCh38 human genome reference (2020-A), UMI counting, sample deconvolution, and TCR clonotype calling. The Seurat toolkit (v5.2.1) was employed in R (v4.4.1) to perform QC, unbiased clustering, dimensionality reduction, and cluster annotation on each aggregated data set. The following QC criteria were enforced to minimize doublets and eliminate low-quality transcriptomes: 1,500 ≥ unique molecular identifier (UMI) count ≤ 20,000; 800 ≥ gene count ≤ 4,400; and mitochondrial UMI percentage ≤ 7%. As we had previously found that MAIT cells are captured among AIM^+^ cells (26), we excluded cells expressing the known MAIT TCRα chain segment TRAV1-2-TRAJ33/12/20, and the TCRβ chain segment TRBV6-1/6-4/20-1 (62). To prevent donor-specific gene expression of individual TCR genes to influence gene expression-based clustering, TCRA/B/D/G genes were pulled from the gene expression matrix and counts were aggregated into a single gene feature for each gene (63). Samples were normalized using the SCTransform function (“V2” regularization, v0.4.2) with mitochondrial content as the variable to regress and 3,000 variable features. Samples were thereafter integrated using the RunHarmony function (v1.2.3), with sequencing batch and donor ID specified as covariates. For CD4 T cells, 20 PCA dimensions were used as cell embedding features, while 15 PCA dimensions were used for the CD8 T cells. FindNeighbors and RunUMAP were performed using 30 dimensions, and FindClusters was performed using a resolution of 0.2 for CD4 T cells and 0.3 for CD8 T cells. The Clustree package (v0.5.1) was used to plot cluster number and cell distribution at different cluster resolutions. The Seurat AddModuleScore function was used to score the CD8 exhaustion gene signature, using 62 genes listed in Table S1, and an average score per donor was calculated.

### Single-cell differential gene expression analysis (DGEA)

The FindAllMarkers function from Seurat (64) (v3.2.3) was used to perform DGEA with MAST (v1.32.0) employed as the statistical framework for differential expression analysis (65). The function was used to identify transcripts enriched in T-cell clusters, as well as to compare the transcriptome of the same cluster between two study groups. Mitochondrial and ribosomal genes were excluded for cluster DGEA between study groups. A gene was considered as differentially expressed if Benjamini-Hochberg adjusted *P* value < 0.05 and either log2 fold change (LFC) ≥ 0.25 or LFC ≤ −0.25. Metascape (66) (http://metascape.org) was used for gene set overrepresentation analyses.

### TCR repertoire analysis

The immunarch R package (v0.9.1) was used to calculate the TCR repertoire diversity of cells with paired TCRα and TCRβ chains. To identify cells with TCR sequences with known antigen-specificity, CDR3 amino acid sequence, V-segment, and J-segments from paired TCRα and TCRβ chain sequences, as well as individual TCRα and TCRβ chains, were mapped to sequences deposited at the VDJ database (Table S6). The scRepertoire (v2.2.1) package was used to visualize the frequency of expansion from paired TCRα and TCRβ clones per cluster and make circle plots showing clonal sharing among clusters.

### Quantification and statistical analysis

Statistical analyses were performed in GraphPad Prism 10.4.2. Data plotted in linear scale were expressed as median ± 95% CI, while the data plotted in logarithmic scales were expressed as geometric mean ± 95% CI. Unpaired comparisons between groups were performed using the nonparametric two-tailed Kruskal-Wallis test adjusted with Dunn’s test for multiple comparisons. The Pearson correlation coefficient test was used for association analysis.

## Data Availability

The scRNA/TCRseq data for the VAX and ABTI donors (GSE297329) and for the SBTI donors (GSE311993) can be found in NCBI Gene Expression Omnibus.

## SUPPLEMENTAL MATERIAL

Supplemental Material mbio.03549-25-s0001.docx Descriptions of Tables S1-S6; Figures S1-S3.

Table S1 mbio.03549-25-s0002.xlsx CD8 exhaustion genes.

Table S2 mbio.03549-25-s0003.xlsx Differential gene expression analyses of group 1 and group 2 CD4 T cell clusters.

Table S3 mbio.03549-25-s0004.xlsx Differential gene expression analyses of TH17-like cells.

Table S4 mbio.03549-25-s0005.xlsx Spike and CD4RE peptides.

Table S5 mbio.03549-25-s0006.xlsx Number of cells specific for non-SARS-CoV-2 epitopes.

Table S6 mbio.03549-25-s0007.xls VDJ database clones.

ASM does not own the copyrights to Supplemental Material that may be linked to, or accessed through, an article. The authors have granted ASM a non-exclusive, world-wide license to publish the Supplemental Material files. Please contact the corresponding author directly for reuse.
