# Supplementary material for: Symptomatic SARS-CoV-2 breakthrough infections broaden the repertoire of Spike-reactive CD4 T cells
Source: mBio. 2025 Dec 12;17(1):e03549-25. doi: 10.1128/mbio.03549-25 (PMC12802171; doi:10.1128/mbio.03549-25)
Supplement: Supplemental Material — Descriptions of Tables S1-S6; Figures S1-S3. [file mbio.03549-25-s0001.docx]

**Supplementary Materials**

**Tables**

**Table S1. CD8 Exhaustion genes**. Genes included in CD8T cell exhaustions signature score.

**Table S2. Differential gene expression analyses of Group 1 and Group 2 CD4 T cell clusters**. Genes expressed at significantly higher or lower levels in Group 1 compared to Group 2 cells (sheet DGEA analysis). Genes expressed at significantly higher levels in Group 1 (sheets “Group 1 enriched genes” and “Group 1 genes – Metascape”) or Group 2 (sheets “Group 2 enriched genes” and “Group 2 genes – Metascape”) were included in gene set overrepresentation analyses.

**Table S3. Differential gene expression analyses of T_H_17-like cells.** Genes expressed at significantly higher or lower levels in T_H_17-like cells in VAX compared to SBTI donors (sheet DGEA analysis). Genes expressed at significantly higher levels in VAX (sheets “Genes upregulated in VAX” and “VAX genes - Metascape”) or SBTI (sheets “Genes upregulated in SBTI” and “SBTI genes - Metascape”) were included in gene set overrepresentation analyses.

**Table S4. Spike and CD4RE peptides**. Peptides included in the SARS-CoV-2 Spike and CD4RE megapools.

**Table S5. Number of cells specific for non-SARS-CoV-2 epitopes.** Description of the peptide sequence and source, and how many cells that were found to be specific for each peptide.

**Table S6. VDJ database clones.** Clones from the VDJ database used to identify previously annotated TCR sequences.

**Figures**

**Figure S1. Single-cell characterization of Spike-specific CD8 T cells.** Dot plot (**A**) and UMAP (**B**) visualization of CD8 cluster marker expression. (**C**) Cluster frequency of all CD8 T cell clusters. Samples were collected from vaccinated donors with no evidence of prior SARS-CoV-2 infection (VAX, n=18), asymptomatic breakthrough infection (ABTI, n=14), and symptomatic BTI (SBTI, n=13) donors. (**D**) Average CD8 exhaustion score per donor. (**E**) Chao1, Gini-Simpson, and D50 (number of clones covering 50% of the repertoire) diversity indexes. P-values from two-tailed Kruskal-Wallis tests followed by Dunn’s multiple comparisons test, and geometric mean ± 95% confidence interval are shown.

**Figure S2. Single-cell characterization of Spike-specific CD4 T cells.** Dot plot (**A**) and UMAP (**B**) visualization of CD4 T cell cluster markers expression. (**C**) Cluster tree visualization of the relationship of cluster number, cell distribution, and clustering resolution. The clusters included in Group 1 and Group 2 are highlighted at resolution 0.2, the resolution used for all analyses. (**D**) Frequency of CD4 clusters. Samples were collected from vaccinated donors with no evidence of prior SARS-CoV-2 infection (VAX, n=18), asymptomatic breakthrough infection (ABTI, n=15), and symptomatic BTI (SBTI, n=13) donors. P-values from two-tailed Kruskal-Wallis tests followed by Dunn’s multiple comparisons test, and geometric mean ± 95% confidence interval are shown.

**Figure S3. Spike-reactive CD4 T cells encompass cytokine producing and responsive cells.** Heatmap (**A**) and UMAP (**B**) projection of genes involved Type II interferon (IFN) signaling and in TCR signaling and proliferation. (**C**) Volcano plot of differentially expressed genes in Cluster 1 – T_H_17 in vaccinated donors with no evidence of prior SARS-CoV-2 infection (VAX) compared to symptomatic breakthrough infection (SBTI) donors. (**D**) TCR alpha CDR3, V-segment, and J-segments previously described to be specific for non-SARS-CoV-2 antigens in the VDJ database, described in **Table S5**. Samples were collected from VAX, (n=18), asymptomatic BTI (ABTI, n=15), and SBTI (n=13) donors.
